# Oxidative Stress as a Mechanistic Link Between Severe Respiratory Viral Infection and Pulmonary Fibrosis

**DOI:** 10.3390/biology15070556

**Published:** 2026-03-31

**Authors:** Shynggys Sergazy, Alexander Gulyaev, Zarina Shulgau

**Affiliations:** LLP “VICTUS PHARM”, Astana 010000, Kazakhstan; akin@mail.ru

**Keywords:** COVID-19, post-viral pulmonary fibrosis, oxidative stress, redox signaling, inflammasome

## Abstract

Post-viral pulmonary fibrosis represents a clinically significant and mechanistically complex consequence of severe respiratory infection. The COVID-19 pandemic has highlighted that a subset of survivors, particularly those with severe pneumonia or acute respiratory distress syndrome, develop persistent fibrosis-like lung abnormalities, including reticulation and traction bronchiectasis, often accompanied by impaired gas transfer. Although the clinical course is heterogeneous and many lesions regress over time, longitudinal studies indicate that structural and functional impairment may persist for years in susceptible individuals. Oxidative stress has emerged as a plausible convergent mechanism linking acute epithelial injury, dysregulated inflammatory resolution, and chronic fibrotic remodeling. Reactive oxygen and nitrogen species amplify inflammatory signaling, promote epithelial cell death and senescence, influence macrophage polarization, and activate canonical profibrotic pathways, notably the TGF-β axis. Redox imbalance is embedded within reinforcing circuits involving NOX4-dependent ROS amplification, mitochondrial dysfunction, endoplasmic reticulum stress, inflammasome activation, and senescence-associated secretory programs. Persistent immune activation and organelle stress may sustain redox dysregulation beyond viral clearance, thereby bridging acute lung injury to maladaptive remodeling. This review integrates epidemiological, clinical, and mechanistic evidence to position oxidative stress as a central mediator of post-viral lung fibrosis and discusses therapeutic and translational implications.

## Simple Summary

Severe respiratory viral infections, including coronavirus disease, can cause long-term damage to the lungs in some patients. While many individuals recover completely, others develop persistent structural changes in lung tissue that resemble pulmonary fibrosis, a condition characterized by scarring and reduced breathing capacity. The reasons why some patients develop long-term lung damage while others recover remain unclear. Increasing evidence suggests that oxidative stress—a harmful imbalance between the production of reactive molecules and the body’s ability to neutralize them—may play an important role in this process. During severe infection, inflammation and cellular injury increase the production of these reactive molecules, which can damage lung cells, impair normal repair mechanisms, and activate pathways that promote scar formation. In some individuals, this imbalance may persist even after the virus has been cleared, contributing to ongoing tissue remodeling and loss of lung function. In this review, we examine clinical observations and biological mechanisms linking oxidative stress to post-viral lung fibrosis and discuss potential therapeutic strategies. Understanding these processes may help identify patients at risk and support the development of treatments that prevent long-term respiratory disability.

## 1. Introduction

Pulmonary fibrosis (PF) is a severe form of interstitial lung disease characterized by dysregulated wound repair, persistent activation and expansion of fibroblasts/myofibroblasts, excessive extracellular matrix deposition, and progressive architectural distortion of the lung parenchyma. Despite advances in antifibrotic therapy for idiopathic pulmonary fibrosis (IPF), PF remains a condition with substantial morbidity and mortality and limited disease-modifying options, especially when triggered by heterogeneous external insults such as viral pneumonia [1,2].

The COVID-19 pandemic has highlighted the capacity of respiratory viral infections to initiate or accelerate fibrotic-like lung remodeling in a subset of patients. While fibrosis was historically considered an uncommon sequela of most viral pneumonias, accumulating evidence from severe influenza, SARS, MERS, and SARS-CoV-2 indicates that extensive epithelial injury, abnormal inflammatory resolution, and maladaptive repair may converge on pro-fibrotic programs [1,3]. Early radiological descriptions of COVID-19 already noted reticulation and other fibrotic-appearing patterns during or shortly after the acute phase [4].

Beyond the acute infection, a major long-term health challenge is the emergence of post-acute sequelae of SARS-CoV-2 infection (PASC), commonly referred to as Long COVID. Population-level modeling and cohort studies indicate that a meaningful proportion of symptomatic infections are followed by persistent symptom clusters, including respiratory manifestations that can substantially impair quality of life [5,6,7]. In patients who were hospitalized with severe COVID-19, persistent parenchymal abnormalities and physiological impairment are common during follow-up, although estimates vary across cohorts and depend on initial severity, imaging protocols, and definitions of fibrosis-like change. A systematic review and meta-analysis that evaluated post-hospitalization CT and lung function across COVID-19 and other viral pneumonitides reported frequent residual parenchymal abnormalities after COVID-19, with a subset showing fibrotic-appearing features months after discharge [8]. Importantly, longer-term follow-up suggests that fibrosis-like abnormalities may persist for years in some survivors of severe disease. In a longitudinal cohort evaluated three years after severe/critical COVID-19, fibrotic-like abnormalities (including reticulations and traction bronchiectasis) were observed in a substantial proportion of participants and were associated with clinical and biological risk factors such as male sex, disease severity, and mechanical ventilation [9]. These observations underline the need to define the mechanistic drivers of post-viral lung fibrosis to support risk stratification, surveillance strategies, and development of targeted interventions.

Among candidate mechanisms, oxidative stress has emerged as a plausible integrative pathway linking persistent injury and inflammation to fibrogenesis. Oxidative stress—broadly defined as an imbalance between reactive oxygen/nitrogen species (ROS/RNS) generation and antioxidant defenses—can amplify pro-inflammatory signaling, promote epithelial cell death and senescence, alter macrophage polarization, and activate canonical pro-fibrotic signaling cascades, particularly the TGF-β axis. In established PF and IPF biology, oxidative stress is increasingly recognized not merely as a bystander but as a driver that interacts with mitochondrial dysfunction, endoplasmic reticulum stress, and profibrotic cellular reprogramming [10]. In the context of Long COVID, multiple mechanistic frameworks propose that persistent immune activation, tissue reservoirs or antigen persistence, dysbiosis, and autoimmunity can sustain redox imbalance and mitochondrial stress over prolonged periods [11,12].

Although this review focuses on post-COVID pulmonary fibrosis, it is important to recognize that different respiratory viruses may initiate fibrotic remodeling through partially overlapping but non-identical injury programs. SARS-CoV-2 infects respiratory epithelial cells through ACE2-dependent mechanisms and targets respiratory epithelial populations, including alveolar type II cells, thereby contributing to lower respiratory tract injury and severe pneumonia [13,14]. In severe disease, this process may be accompanied by diffuse alveolar damage, endothelial dysfunction, complement-associated vascular injury, and prolonged immune activation, all of which may favor persistent tissue remodeling [15]. By contrast, influenza viruses and earlier pathogenic coronaviruses such as SARS-CoV and MERS-CoV also induce severe epithelial injury and ARDS, but differ in host–cell tropism, pathogenic behavior, and the relative contribution of direct cytopathic damage versus secondary inflammatory injury. These distinctions may influence the intensity and persistence of oxidative stress, the quality of epithelial repair, and the likelihood of fibrosis-like remodeling after recovery [16].

**Figure 1:**
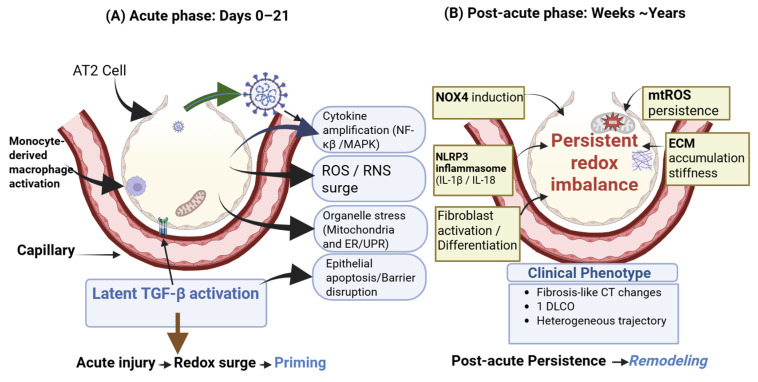
Proposed redox-centered model linking acute SARS-CoV-2 lung injury to post-viral fibrotic remodeling. (**A**) During acute severe COVID-19, epithelial injury, cytokine amplification, and organelle stress drive ROS/RNS production and activation of profibrotic signaling. (**B**) In susceptible individuals, persistent immune activation and organelle dysfunction may sustain redox imbalance, reinforcing TGF-β/NOX4 signaling, inflammasome activity, and senescence programs that promote fibroblast persistence and extracellular matrix remodeling, resulting in fibrosis-like abnormalities and impaired gas transfer.

Given the clinical burden of post-COVID respiratory sequelae and the plausibility of oxidative stress as a convergent mechanism across acute viral injury, chronic immune dysregulation, and fibrotic remodeling, a focused synthesis is timely. In this review, we summarize clinical evidence for post-viral (with emphasis on post-COVID) fibrotic-like lung abnormalities and discuss mechanistic links between persistent oxidative stress and core fibrogenic programs, including epithelial dysfunction, macrophage-driven profibrotic signaling, and extracellular matrix remodeling. We further highlight knowledge gaps and priorities for translational research aimed at validating redox-related biomarkers and testing redox-modulating strategies in post-viral pulmonary fibrosis. Figure 1 provides an overview of the proposed redox-centered trajectory from acute injury to persistent remodeling.

## 2. Epidemiology and Clinical Evidence of Post-COVID Pulmonary Fibrosis

### 2.1. Radiological Sequelae After Acute COVID-19

Persistent radiological abnormalities are frequently reported following hospitalization for COVID-19 pneumonia. Early observational studies demonstrated that even after viral clearance and clinical stabilization, computed tomography (CT) often revealed residual ground-glass opacities, reticulation, parenchymal bands, and traction bronchiectasis [4,17]. These findings raised concern that a subset of patients might develop progressive fibrotic remodeling rather than reversible post-inflammatory changes.

A comprehensive systematic review and meta-analysis by Fabbri et al., which evaluated parenchymal lung abnormalities after hospitalization for COVID-19 and other viral pneumonias, reported that at a median follow-up of approximately three months, inflammatory changes were present in ~50% of patients and fibrotic-appearing abnormalities in ~29%. Importantly, impaired gas transfer was observed in ~38% of individuals, indicating that radiological findings were frequently accompanied by functional impairment [8]. Estimates vary by follow-up interval and radiological definitions. These data suggest that post-viral structural abnormalities are not rare and may have clinically meaningful physiological consequences.

Longer follow-up studies have provided additional insight. In a six-month follow-up cohort of patients with severe COVID-19 pneumonia, persistent CT abnormalities were common, particularly in individuals who required intensive care [17]. One-year longitudinal analyses have confirmed that a proportion of patients continue to demonstrate structural abnormalities and reduced diffusing capacity (DLCO), even when overt symptoms have improved [18]. Although many lesions gradually regress, a subset exhibits stable or slowly evolving fibrosis-like patterns.

### 2.2. Three-Year Outcomes and Persistent Fibrosis-like Abnormalities

Long-term data extending beyond one year remain limited but are emerging. In a longitudinal multiethnic cohort study evaluating survivors three years after severe or critical COVID-19, Murphy et al. reported that fibrotic-like abnormalities—including reticulation and traction bronchiectasis—were present in approximately 61% of participants. In adjusted analyses, these abnormalities were associated with male sex, shorter leukocyte telomere length, greater initial disease severity, and mechanical ventilation during the acute phase [9]. These findings indicate that severe SARS-CoV-2 infection may initiate durable remodeling processes in a substantial subset of patients.

Importantly, not all radiological fibrosis-like findings necessarily correspond to progressive idiopathic pulmonary fibrosis. High-resolution computed tomography (HRCT) remains the cornerstone for identifying and characterizing suspected pulmonary fibrosis, as it defines the distribution and pattern of interstitial abnormalities and helps distinguish fibrotic changes from predominantly inflammatory or potentially reversible post-infectious changes. At the same time, HRCT findings must be interpreted cautiously, since similar radiologic patterns may also occur in connective tissue disease–associated interstitial lung disease, organizing pneumonia, drug-related lung injury, and other non-idiopathic interstitial processes. Accordingly, imaging should be integrated with clinical history, serologic evaluation, pulmonary function testing, and multidisciplinary discussion to support accurate differential diagnosis and appropriate follow-up [19,20,21].

### 2.3. Functional Impairment and Clinical Correlates

Functional impairment often parallels structural abnormalities. Reduced DLCO is one of the most consistently reported physiological abnormalities after severe COVID-19 [8,17]. Restrictive ventilatory defects are less common but may be observed in patients with extensive parenchymal involvement. The persistence of diffusion impairment suggests ongoing microvascular or interstitial pathology.

Acute respiratory distress syndrome (ARDS) during hospitalization is one of the strongest predictors of long-term fibrotic sequelae. Patients requiring invasive mechanical ventilation exhibit higher rates of residual abnormalities compared with those managed with non-invasive support [1]. Age, male sex, prolonged ICU stay, and high inflammatory burden during acute infection have also been associated with persistent parenchymal changes [9].

Beyond hospitalized cohorts, epidemiological modeling indicates that respiratory symptom clusters remain common within the broader Long COVID population [5]. While not all persistent dyspnea reflects fibrosis, the overlap between symptom clusters and radiological abnormalities underscores the need for structured long-term pulmonary follow-up.

### 2.4. Comparison with Other Viral Pneumonias

The association between viral infection and fibrotic remodeling is not unique to SARS-CoV-2. Post-SARS and post-MERS cohorts demonstrated persistent radiological and functional abnormalities months to years after infection [1]. Severe influenza pneumonia has similarly been linked to subsequent fibrotic remodeling in a subset of patients. These precedents support the concept that intense epithelial injury combined with dysregulated repair may trigger maladaptive fibrosis programs following viral pneumonia.

Taken together, epidemiological and longitudinal data indicate that fibrosis-like abnormalities are relatively common after severe COVID-19, frequently accompanied by impaired gas transfer. Disease severity and ARDS represent major risk factors, and a subset of patients exhibits persistent abnormalities extending for several years. These observations provide a clinical framework for investigating mechanistic drivers of post-viral pulmonary fibrosis, including the potential role of sustained oxidative stress and redox imbalance. Key cohort characteristics, follow-up duration, imaging findings, functional outcomes, and major risk factors are summarized in Table 1. While these epidemiological data establish the clinical relevance of post-COVID fibrotic-like abnormalities, understanding the underlying biological mechanisms requires a closer examination of core pathways driving pulmonary fibrogenesis. These clinical patterns raise a mechanistic question: which injury–repair programs persist after viral clearance and bias the lung toward maladaptive remodeling?

## 3. General Mechanisms of Pulmonary Fibrosis

Pulmonary fibrosis is increasingly understood as a maladaptive wound-healing response in which severe or repetitive epithelial injury fails to resolve appropriately, leading to persistent fibroblast activation, extracellular matrix (ECM) accumulation, and progressive architectural distortion of the lung. Rather than representing a linear cascade, contemporary models describe fibrogenesis as a dynamic, multicellular network involving epithelial cells, mesenchymal populations, immune cells, and the extracellular matrix itself [22].

The process is typically initiated by injury to alveolar epithelial type II (AT2) cells, which serve as progenitors responsible for maintaining alveolar integrity. In idiopathic pulmonary fibrosis (IPF) and other fibrotic lung diseases, epithelial cells exhibit impaired regenerative capacity, altered differentiation trajectories, and stress-associated phenotypes characterized by the secretion of profibrotic mediators [23]. Instead of restoring normal architecture, the injured epithelium generates a microenvironment enriched in transforming growth factor-β (TGF-β), chemokines, and damage-associated molecular patterns that recruit and activate fibroblasts and immune cells.

Fibroblast activation and myofibroblast differentiation represent the central effector phase of fibrosis. Under the influence of TGF-β and other mediators, fibroblasts acquire contractile properties, increase collagen synthesis, and remodel the ECM [24]. As matrix stiffness increases, mechanotransduction pathways further reinforce fibroblast activation, creating a self-sustaining loop in which structural remodeling perpetuates cellular reprogramming [22].

Immune–mesenchymal crosstalk plays a critical modulatory role. Macrophages integrate epithelial injury signals and adopt context-dependent phenotypes that can either promote resolution or drive fibrosis. Profibrotic macrophage populations secrete TGF-β and additional growth factors that stimulate fibroblast proliferation and differentiation [25,26]. Sustained inflammatory signaling thereby stabilizes mesenchymal activation and ECM accumulation.

Inflammatory amplification circuits further embed fibrosis within chronic tissue remodeling. Epithelial plasticity programs, including partial epithelial–mesenchymal transition (EMT)-like states, contribute to barrier dysfunction and enhanced responsiveness to TGF-β [23]. Concurrently, activation of the NLRP3 inflammasome promotes IL-1β–driven inflammatory signaling that enhances fibroblast activation and matrix deposition [27].

Cellular senescence provides an additional stabilizing mechanism. Senescent epithelial cells and fibroblasts acquire a senescence-associated secretory phenotype (SASP), characterized by secretion of IL-6, TGF-β, and other profibrotic mediators [22]. This milieu reinforces fibroblast persistence and limits regenerative capacity, particularly in aging lungs. Collectively, these processes define pulmonary fibrosis as a network disease in which epithelial dysfunction, immune activation, and mesenchymal reprogramming converge to sustain structural remodeling.

## 4. Oxidative Stress in Acute COVID-19

Severe SARS-CoV-2 infection is characterized by profound disruption of immune and redox homeostasis. Beyond direct viral cytopathic effects, acute COVID-19 frequently involves excessive inflammatory activation and oxidative imbalance, particularly in patients who develop acute respiratory distress syndrome (ARDS) [28].

Reactive oxygen species (ROS) and reactive nitrogen species (RNS) are tightly intertwined with inflammatory signaling cascades. During severe COVID-19, elevated cytokine production—often described as a cytokine storm—activates NF-κB and MAPK pathways that both generate and are amplified by oxidative stress. ROS enhances cytokine transcription, while inflammatory mediators further stimulate ROS production, creating a self-reinforcing inflammatory–redox loop [28]. Meta-analytical evidence demonstrates a significant association between oxidative stress markers and disease severity, supporting the concept that redox imbalance correlates with clinical deterioration [29].

At the cellular level, organelle dysfunction amplifies oxidative stress. Mitochondria, central regulators of cellular metabolism and innate immune signaling, become functionally impaired during SARS-CoV-2 infection. Disrupted oxidative phosphorylation and metabolic reprogramming contribute to sustained mitochondrial ROS generation and altered antiviral responses [10,30]. Concurrently, endoplasmic reticulum (ER) stress activates the unfolded protein response (UPR), which is closely linked to oxidative pathways and apoptosis [31,32]. Crosstalk between mitochondrial dysfunction and ER stress further destabilizes cellular homeostasis and promotes epithelial injury. This interaction is increasingly understood through mitochondria-associated ER membranes (MAMs), which coordinate calcium transfer, redox signaling, apoptotic susceptibility, and metabolic adaptation. In fibrotic lung disease, disruption of ER–mitochondrial tethering has been linked to alveolar epithelial cell stress and apoptosis, suggesting that organelle contact sites may function as an emerging mechanistic hub connecting proteostasis failure to redox-driven remodeling [33].

Importantly, many molecular programs activated during acute COVID-19 overlap with established fibrotic pathways. ROS can activate TGF-β signaling, enhance fibroblast differentiation, stimulate inflammasome activation, and promote epithelial apoptosis. In the setting of ARDS—a recognized risk factor for subsequent pulmonary fibrosis—marked oxidative imbalance may therefore function as an early priming event that predisposes susceptible individuals to persistent remodeling [1]. Acute redox dysregulation may thus represent the first step in a trajectory that transitions from inflammatory injury to chronic fibrotic change.

## 5. Persistent Oxidative Stress in Long COVID

While oxidative stress is well documented during acute SARS-CoV-2 infection, an unresolved question is whether redox disturbances fully resolve after viral clearance or persist in a subset of individuals with Long COVID. Emerging evidence suggests that persistent immune activation, metabolic dysregulation, and mitochondrial stress may sustain redox imbalance beyond the acute phase [11,12].

Chronic low-grade inflammation represents a plausible driver of sustained ROS production. Prolonged activation of innate and adaptive immune cells can maintain oxidative signaling even in the absence of active viral replication. Reviews of post-COVID pulmonary sequelae emphasize that incomplete resolution of tissue injury may create a microenvironment conducive to continued redox imbalance [34]. Mitochondrial dysfunction, frequently described in post-viral syndromes, may further perpetuate ROS generation and impair epithelial recovery [10,22].

Chronic oxidative stress is not merely a marker of persistent inflammation but an active participant in fibrogenic signaling. ROS interact bidirectionally with TGF-β pathways, promote myofibroblast differentiation, and enhance extracellular matrix deposition [35,36]. In established fibrosis, sustained NOX4-dependent ROS production reinforces fibroblast activation, while mitochondrial dysfunction, inflammasome activation, and senescence create a profibrotic microenvironment [22,27].

In this framework, persistent redox dysregulation may serve as a mechanistic bridge linking acute SARS-CoV-2–induced injury to long-term fibrotic remodeling. Individuals with severe acute lung injury, ARDS, genetic susceptibility, or age-related telomere shortening may be particularly vulnerable to this transition. Although definitive longitudinal studies correlating oxidative stress biomarkers with structural lung remodeling remain limited, the mechanistic convergence between Long COVID biology and established fibrosis pathways strongly supports further investigation of sustained oxidative stress as a driver of post-viral pulmonary fibrosis.

## 6. Molecular Crosstalk Between Oxidative Stress and Fibrogenic Signaling

Pulmonary fibrogenesis represents a dynamic and self-reinforcing network in which epithelial injury, immune activation, fibroblast reprogramming, and extracellular matrix (ECM) deposition are tightly interconnected. Within this network, oxidative stress functions not merely as a secondary consequence of inflammation but as a systems-level integrator that amplifies and stabilizes profibrotic signaling circuits [10,35].

A central axis of this integration involves the bidirectional interaction between reactive oxygen species (ROS) and transforming growth factor-β (TGF-β), widely regarded as the master regulator of pulmonary fibrosis. ROS can activate latent TGF-β stored in the extracellular matrix, thereby enhancing downstream SMAD2/3 signaling and promoting myofibroblast differentiation [35]. In parallel, TGF-β induces intracellular ROS production, particularly via NADPH oxidase isoforms such as NOX4, establishing a positive feedback loop that sustains fibroblast activation and collagen synthesis [10,36]. In addition to canonical SMAD2/3 signaling, TGF-β may also activate non-canonical pathways, including MAPK, PI3K/AKT, and Rho/ROCK signaling, which further contribute to fibroblast activation and extracellular matrix remodeling [22,24]. This reciprocal reinforcement contributes to the persistence of myofibroblast phenotypes even after resolution of the initial injurious stimulus.

Among redox-generating systems, NOX4 has emerged as a critical effector of fibrotic remodeling. Unlike transient ROS bursts associated with acute inflammatory responses, NOX4-mediated ROS production is relatively sustained and closely coupled to TGF-β signaling [36]. Persistent NOX4 activity supports ECM accumulation, enhances contractile properties of fibroblasts, and promotes matrix stiffening, thereby reinforcing mechanotransduction pathways that further stimulate fibrogenesis. In this context, redox dysregulation becomes embedded within the structural remodeling process itself.

Mitochondrial dysfunction provides an additional layer of redox–fibrotic crosstalk. In fibrotic lung tissue, impaired mitochondrial bioenergetics and excessive mitochondrial ROS (mtROS) generation contribute to epithelial apoptosis, impaired regenerative capacity of alveolar type II cells, and activation of downstream inflammatory pathways [22]. Mitochondrial ROS can potentiate TGF-β signaling and facilitate activation of the NLRP3 inflammasome, thereby linking organelle stress with both inflammatory amplification and mesenchymal reprogramming [10,27]. In this manner, mitochondrial dysfunction serves as a bridge connecting acute injury to chronic structural remodeling.

Inflammasome activation represents another redox-sensitive node within fibrotic networks. Reactive oxygen species are well-established triggers of NLRP3 inflammasome activation, leading to maturation and secretion of IL-1β and IL-18 [27]. Sustained inflammasome signaling perpetuates inflammatory recruitment, promotes fibroblast activation, and enhances ECM deposition, thereby embedding innate immune activation within the fibrotic cascade. Importantly, ROS-driven inflammasome activation further amplifies oxidative stress, creating an additional feedback loop.

Chronic oxidative stress also promotes cellular senescence, a state characterized by irreversible cell cycle arrest and acquisition of a senescence-associated secretory phenotype (SASP). Senescent epithelial cells and fibroblasts secrete proinflammatory and profibrotic mediators, including TGF-β and IL-6, thereby reinforcing fibroblast activation and matrix remodeling [22]. Oxidative DNA damage and p53/p21 activation link redox imbalance directly to senescence induction, while SASP components further enhance oxidative stress, creating a feed-forward circuit that limits regenerative capacity and stabilizes fibrotic architecture.

Emerging evidence also suggests that oxidative stress may intersect with epigenetic and transcriptomic remodeling in pulmonary fibrosis. Redox imbalance can influence DNA methylation, histone modification, and non-coding RNA expression, thereby stabilizing profibrotic transcriptional programs even after the initial injury has subsided. Multiomic analyses of idiopathic pulmonary fibrosis have identified coordinated changes across the transcriptome, methylome, proteome, and long non-coding RNA landscape, supporting the concept that epigenetic regulation contributes to persistent fibrogenic reprogramming. In parallel, circulating coding and long non-coding RNAs have been proposed as potential noninvasive biomarkers of fibrotic lung disease, raising the possibility that redox-sensitive molecular signatures may eventually complement imaging-based assessment and improve patient stratification [37,38].

Collectively, these interconnected pathways illustrate how oxidative stress integrates multiple profibrotic mechanisms into a coherent amplification network. ROS activate TGF-β signaling; TGF-β induces NOX4-dependent ROS generation; mitochondrial dysfunction sustains redox imbalance and epithelial apoptosis; inflammasome activation reinforces inflammatory–fibrotic coupling; and senescence establishes a chronic profibrotic microenvironment. Rather than functioning as an isolated biochemical abnormality, oxidative stress emerges as a central organizing principle that stabilizes and propagates fibrotic remodeling [10,35]. A structured overview of key redox-sensitive molecular pathways is provided in Table 2. This network-based view suggests that effective interventions may need to target defined redox nodes rather than broadly scavenge reactive species.

## 7. Therapeutic Targeting of Oxidative Stress in Post-Viral Pulmonary Fibrosis

The central positioning of oxidative stress within fibrogenic signaling networks provides a compelling rationale for therapeutic intervention. However, attempts to modulate redox imbalance in pulmonary fibrosis have historically yielded mixed results, highlighting the complexity of oxidative biology in chronic lung disease. In the context of post-viral pulmonary fibrosis, including post-COVID remodeling, therapeutic strategies must account for the distinction between transient oxidative bursts during acute inflammation and sustained, signaling-driven redox amplification that perpetuates fibrotic pathways.

Initial efforts to target oxidative stress focused on broad antioxidant supplementation. N-acetylcysteine (NAC), a precursor of glutathione, was evaluated in idiopathic pulmonary fibrosis (IPF) with the aim of restoring intracellular antioxidant capacity. Although early studies suggested potential benefit, the PANTHER-IPF trial demonstrated that NAC monotherapy did not significantly improve clinical outcomes in unselected IPF populations [40,41]. These findings underscored a critical concept: simple ROS scavenging may be insufficient when oxidative stress is embedded within self-reinforcing signaling networks rather than representing a purely quantitative excess of reactive species. Nevertheless, NAC studies remain mechanistically informative, reinforcing the role of impaired glutathione homeostasis in fibrotic lungs [35].

More targeted approaches aim to inhibit enzymatic sources of pathogenic ROS production. Among these, NOX4 has emerged as a particularly attractive candidate. As a downstream effector of TGF-β signaling and a driver of sustained ROS generation in fibroblasts, NOX4 integrates redox amplification with myofibroblast differentiation and extracellular matrix deposition [36]. Experimental inhibition of NOX4 attenuates fibrotic remodeling in preclinical models, supporting the concept that targeting upstream ROS-generating enzymes may be more effective than broad antioxidant therapy [10]. Although definitive clinical translation remains pending, this strategy directly addresses a mechanistic node central to redox-driven fibrosis.

Given the prominent role of mitochondrial dysfunction in fibrotic lung disease, organelle-targeted antioxidants represent another promising avenue. Mitochondria-targeted compounds such as MitoQ and related molecules aim to selectively reduce mitochondrial ROS production and restore bioenergetic stability. Preclinical models suggest that modulation of mitochondrial redox balance can attenuate epithelial apoptosis and downstream fibrotic signaling [22]. In post-viral contexts, where mitochondrial stress may persist beyond viral clearance, such targeted approaches could theoretically interrupt the transition from acute injury to chronic remodeling.

Importantly, not all redox-directed strategies act upstream at the level of ROS production. Several therapeutic concepts focus on downstream pathways that are highly sensitive to oxidative stress. The NLRP3 inflammasome, a redox-responsive inflammatory complex, represents one such target. ROS-mediated inflammasome activation sustains IL-1β production and promotes fibroblast activation [27]. Pharmacologic modulation of inflammasome signaling may therefore attenuate oxidative–inflammatory amplification without directly scavenging reactive species.

Similarly, cellular senescence has emerged as a redox-amplified state that reinforces fibrotic progression. Oxidative stress promotes DNA damage and senescence, while senescent cells secrete profibrotic mediators through the senescence-associated secretory phenotype (SASP) [22]. Senolytic strategies, although still largely experimental in pulmonary fibrosis, represent an innovative attempt to disrupt this feed-forward loop between redox imbalance and persistent fibroblast activation.

Current antifibrotic agents approved for IPF, including nintedanib and pirfenidone, do not function primarily as antioxidants but intersect with redox-related pathways. Pirfenidone exhibits anti-TGF-β and partial antioxidant properties in experimental systems [35], whereas nintedanib inhibits multiple receptor tyrosine kinases involved in fibroblast proliferation and migration. Importantly, nintedanib has demonstrated benefit in progressive fibrosing interstitial lung diseases beyond IPF [42], raising the possibility that similar agents may have utility in selected patients with post-viral fibrotic phenotypes. However, robust randomized trials specifically targeting post-COVID fibrosis remain limited. Recent clinical evidence remains preliminary, including observational data for nintedanib and the phase 2 FIBRO-COVID trial of pirfenidone, which did not show a statistically significant advantage over placebo in the overall improvement of lung function or HRCT fibrotic score after 6 months [43,44].

Taken together, therapeutic targeting of the redox–fibrotic axis requires a shift from nonspecific antioxidant supplementation toward mechanistically informed strategies that interrupt defined amplification nodes within fibrogenic networks. Future clinical studies may benefit from biomarker-guided patient stratification, identification of redox-defined endotypes, and combination approaches that simultaneously modulate upstream oxidative drivers and downstream profibrotic signaling pathways.

## 8. Knowledge Gaps and Future Directions

Despite growing recognition of oxidative stress as a key integrator of fibrogenic signaling, several critical gaps remain in our understanding of post-viral pulmonary fibrosis, particularly in the context of COVID-19.

First, direct longitudinal studies linking persistent oxidative stress biomarkers to structural lung remodeling in post-COVID cohorts are lacking. While redox imbalance has been well characterized in idiopathic pulmonary fibrosis and acute lung injury [10,35], comparable prospective data in Long COVID populations remain scarce. The absence of standardized oxidative stress biomarkers limits risk stratification and mechanistic validation. Candidate markers that may help bridge mechanistic and clinical assessment include circulating epithelial injury markers such as KL-6 and SP-D, as well as oxidative stress readouts such as 8-isoprostane, although none are currently validated as stand-alone predictors of post-viral fibrotic progression. Serial high-resolution CT and diffusing capacity measurements may provide complementary structural and functional correlates for biomarker-based risk stratification [45,46,47].

Second, it remains unclear whether persistent oxidative stress is a primary driver of fibrosis progression or a secondary amplifier of upstream immune dysregulation. Distinguishing causation from association requires well-designed mechanistic studies integrating redox profiling, imaging, lung function testing, and molecular phenotyping.

Third, patient heterogeneity presents a major challenge. Only a subset of individuals with severe COVID-19 develop long-term fibrosis-like abnormalities. A clinically useful stratification framework will likely need to integrate acute disease severity, ARDS/mechanical ventilation exposure, imaging pattern, pulmonary function trajectory, and host susceptibility factors such as age or telomere-related vulnerability. In this context, combining radiological follow-up with molecular markers of epithelial injury and redox imbalance may help define higher-risk endotypes more suitable for targeted intervention [9,22].

Fourth, therapeutic translation remains underdeveloped. Although redox-modulating strategies show promise in preclinical fibrosis models, clinical trials specifically targeting oxidative stress in post-viral pulmonary fibrosis are minimal. Future studies should incorporate biomarker-guided enrollment and combination approaches targeting both upstream redox amplification and downstream fibrotic signaling.

Finally, experimental models of post-viral fibrotic remodeling require refinement. Current fibrosis models (e.g., bleomycin-induced injury) do not fully recapitulate the immunological and viral persistence features observed in post-COVID states. Development of virus-informed models may clarify the temporal relationship between oxidative stress persistence and fibrotic transition.

Addressing these gaps will be critical to determining whether oxidative stress represents a viable therapeutic target or a parallel epiphenomenon in post-viral lung remodeling.

## 9. Conclusions

Pulmonary fibrosis following severe viral infection represents a complex, multi-layered process involving epithelial injury, immune dysregulation, fibroblast activation, and extracellular matrix remodeling. Within this network, oxidative stress emerges as a central integrative amplifier rather than a passive consequence of inflammation.

Reactive oxygen species interact bidirectionally with TGF-β signaling, sustain NOX4-dependent fibroblast activation, promote mitochondrial dysfunction, activate inflammasomes, and induce cellular senescence. These interconnected processes create a self-reinforcing redox–fibrotic network capable of perpetuating structural remodeling beyond the resolution of acute infection.

In the context of COVID-19, the persistence of oxidative and mitochondrial stress may help explain why a subset of patients develops long-term fibrosis-like abnormalities after severe disease. Although direct longitudinal evidence remains limited, the mechanistic convergence between established pulmonary fibrosis biology and post-viral immune dysregulation provides a coherent conceptual framework. Future translational efforts should prioritize the identification of redox-based biomarkers, mechanistic endotyping of patients, development of virus-informed fibrosis models, and rigorous evaluation of targeted redox-modulating therapies.

Clarifying the role of oxidative stress in post-viral pulmonary fibrosis may ultimately open new avenues for early intervention and prevention of chronic structural lung damage.

## Figures and Tables

**Table 1:** Key cohort and meta-analytical studies reporting fibrosis-like lung abnormalities after COVID-19.

Population and Severity	Follow-Up Duration	Imaging Findings (Fibrosis-like Changes)	Functional Impairment	Major Risk Factors Identified	Reference
Hospitalized COVID-19 patients (mixed severity)	Acute phase	Early reticulation, parenchymal bands, fibrotic-appearing changes observed in a subset	Not primary endpoint	Greater radiologic involvement in severe cases	[4]
Severe COVID-19 pneumonia (hospitalized)	6 months	Persistent CT abnormalities; reticulation and traction bronchiectasis in subset	Reduced DLCO common; diffusion impairment frequent	ICU admission; higher initial lung involvement	[17]
Post-hospitalized COVID-19 and other viral pneumonitis (meta-analysis, multiple cohorts)	Median ~3 months	~29% fibrotic-appearing abnormalities; ~50% inflammatory changes	~38% abnormal lung function (impaired gas transfer)	Severe initial disease; hospitalization	[8]
Post-COVID hospitalized patients	12 months	Persistent structural abnormalities in subset	Reduced DLCO at 1 year; occasional restrictive defects	Acute disease severity	[18]
Survivors of severe/critical COVID-19 (multiethnic cohort)	3 years	61% fibrosis-like abnormalities (reticulation, traction bronchiectasis)	Persistent diffusion impairment in subset	Male sex; mechanical ventilation; higher severity; shorter leukocyte telomere length	[9]
Severe COVID-19 and ARDS (review of post-ARDS remodeling)	Variable	ARDS-associated fibrotic remodeling discussed	Post-ARDS restrictive physiology described	ARDS; mechanical ventilation	[1]
Global Long COVID population modeling	1–2 years (modeled)	Not imaging-based; respiratory symptom cluster common	Persistent respiratory symptoms	Symptomatic infection; greater severity increases persistence	[5]

**Table 2:** Redox-sensitive molecular pathways implicated in pulmonary fibrogenesis.

Redox-Associated Mechanism	Key Molecular Components	Cellular Targets	Profibrotic Consequence	Reference
ROS-mediated activation of TGF-β signaling	ROS, TGF-β1, Smad2/3	Epithelial cells, fibroblasts	Myofibroblast differentiation, ECM deposition	[35]
NOX4-dependent ROS amplification	NOX4, NADPH oxidase	Fibroblasts	Sustained TGF-β signaling, collagen synthesis	[36]
Mitochondrial dysfunction and mtROS release	mtROS, cytochrome c, mtDNA	Epithelial cells, macrophages	Epithelial apoptosis, inflammatory amplification	[10]
NLRP3 inflammasome activation	ROS, NLRP3, IL-1β, IL-18	Macrophages	Chronic inflammation, fibroblast activation	[27]
Oxidative stress–induced EMT	ROS, TGF-β, Wnt/β-catenin	Alveolar epithelial cells	Fibroblast-like phenotype transition	[23]
Senescence and SASP amplification	ROS, p53, p21, IL-6, TGF-β	Epithelial cells, fibroblasts	Persistent profibrotic microenvironment	[22]
ER stress–redox crosstalk	UPR, CHOP, PERK, ROS	Epithelial cells	Apoptosis, maladaptive repair	[31]
Ferroptosis and lipid peroxidation	Lipid ROS, GPX4, iron	Epithelial cells	Enhanced fibrotic remodeling	[39]

## Data Availability

No new data were created or analyzed in this study. Data sharing is not applicable to this article.
